# 

**Published:** 1887-05-07

**Authors:** 


PRICE ONE PENNY.

No. 32, Vol. II.

May 7th, 1887.

[Registered at the General Post Office as a Newspaper.]

Per Annum, 6s. 6d., post free.
Monthly Parts, 6d.; post free, 7½d.
Ditto per Ann., 7s. 6d., post free.

